# Small‐Scale Field Trials to Assess the Molluscicidal Activity of *Hagenia abyssinica* Against *Biomphalaria* Snail Species

**DOI:** 10.1155/japr/1322550

**Published:** 2026-06-17

**Authors:** Hirut Basha, Asfaw Debella, Milkyas Endale, Meharu Mathewos, Habtamu Tadesse, Hassen Mamo

**Affiliations:** ^1^ Traditional and Modern Medicine Research and Development Directorate, Armauer Hansen Research Institute, Addis Ababa, Ethiopia, ahri.gov.et; ^2^ National Fisheries and Aquatic Life Research Center, Ethiopian Institute of Agricultural Research, Sebeta, Ethiopia, eiar.gov; ^3^ Department of Microbial Sciences and Genetics, Addis Ababa University, Addis Ababa, Ethiopia, aau.edu.et

**Keywords:** *Biomphalaria*, field trials, fish toxicity, *Hagenia abyssinica*

## Abstract

Schistosomiasis remains a significant public health burden in many developing countries. Plant‐based molluscicides have gained considerable attention as alternatives for snail control, owing to their relatively lower toxicity and favorable environmental profile. However, prior to large‐scale application, plant‐based molluscicides require rigorous small‐scale field trials and comprehensive toxicity assessments under natural conditions to confirm their efficacy and evaluate their potential impact on nontarget organisms, particularly fish. This study evaluated the molluscicidal activity of 70% ethanol and chloroform fractions of *Hagenia abyssinica* flowers against *Biomphalaria* species and assessed the acute toxicity of the 70% ethanol extract on Nile tilapia (*Oreochromis niloticus*) through a small‐scale trial. Female flowers of *H. abyssinica* were collected in Addis Ababa, Ethiopia. Adult *Biomphalaria* species were exposed to 70% ethanol and chloroform fractions of *H. abyssinica* for 24, 48, and 72 h. Fish toxicity was assessed following the Organisation for Economic Co‐operation and Development guidelines at the National Fish and Other Aquatic Lives Research Center in Sebeta. Lethal concentration values (LC_50_ and LC_90_) were calculated from mortality data using probit regression analysis in IBM SPSS software. In fish toxicity assessments, the LC_50_ and LC_90_ for the 70% ethanol extract were 38.71 and 57.14 mg/L, respectively. Fish fingerlings showed no significant difference in survival at concentrations up to 16 mg/L, establishing this level as the no observed adverse effect concentration (NOAEC). The study found that both the 70% ethanol extract and chloroform fraction of *H. abyssinica* flowers displayed significant molluscicidal activity against *Biomphalaria* species, with LC_50_ values of 78.77 and 36.49 mg/L, respectively, after 24 h. The LC_50_ of the 70% ethanol extract for molluscicidal activity was about twice that needed for fish, showing that fish were more sensitive than *Biomphalaria* snails. This differential toxicity raises important ecological concerns.

## 1. Background

Schistosomiasis is a major public health problem, particularly in Africa, South America, the Caribbean, and the Eastern Mediterranean [1–4], with Africa accounting for 93% of the estimated 207 million schistosomiasis cases globally [5]. In support of the global goal to eliminate schistosomiasis by 2030. The World Health Organization advocates integrated control strategies that include the use of chemical molluscicides to target intermediate‐host snails. Nonetheless, chemical molluscicides have several limitations, including toxicity to nontarget organisms, high costs, and adverse environmental effects [6, 7]. In addition, resistance to commonly used molluscicides has been reported in some snail populations [8].

To address these challenges, the use of medicinal plants for snail control has been widely recommended, as plant‐based molluscicides are generally less toxic, more cost‐effective, and environmentally sustainable [9]. According to the WHO criteria, a plant is considered a promising laboratory molluscicide if it achieves ≥ 90% snail mortality within 24 h at a concentration not exceeding 100 mg/L [10]. Such a potential candidate should undergo field trials to assess efficacy under natural ecological conditions. In our previous laboratory study, 70% ethanol extract and the chloroform fraction of *H. abyssinica* flowers showed significant molluscicidal activities against *Biomphalaria* species, with 24‐h LC_50_ values of 11.93 and 5.52 mg/L, respectively [11], justifying further field evaluations. Additionally, before field application, it is imperative to assess the potential toxicological effects of these extracts on nontarget organisms, particularly fish, to ensure environmental safety and ecological integrity. To date, the acute fish toxicity of this promising molluscicide plant has not been reported. Therefore, this study is aimed at conducting small‐scale field trials to assess the molluscicidal activity of 70% ethanol extract and chloroform fractions of *H. abyssinica* flowers against *Biomphalaria* species, and evaluating the acute toxicity of the 70% ethanol extract on Nile tilapia fish, *Oreochromis niloticus*.

This study generates new knowledge on the molluscicidal activity of *H. abyssinica* against schistosomiasis‐transmitting snails, contributing to the discovery of safer and more cost‐effective alternatives to synthetic molluscicides such as niclosamide. Furthermore, it provides critical information on the safety profiles of *H. abyssinica* extracts toward nontarget organisms, particularly fish, thereby supporting its potential for environmentally responsible field application.

## 2. Materials and Methods

### 2.1. About the Plant: Collection, Preparation, and Extraction

*H. abyssinica* (Bruce) J. F. Gmel., called Kousso or Kosso in East and Central Africa, is the only species in the genus *Hagenia* (Rosaceae) [12]. This dioecious tree, with separate male and female plants, is a characteristic component of Afro‐Montane Forest ecosystems across Central and Eastern Africa [12]. It typically grows at altitudes between 2000 and 3000 m above sea level [13]. The flowers of *H. abyssinica* are notable for their antiparasitic properties, particularly in the treatment of tapeworm infestations [14].

**Figure 1:**
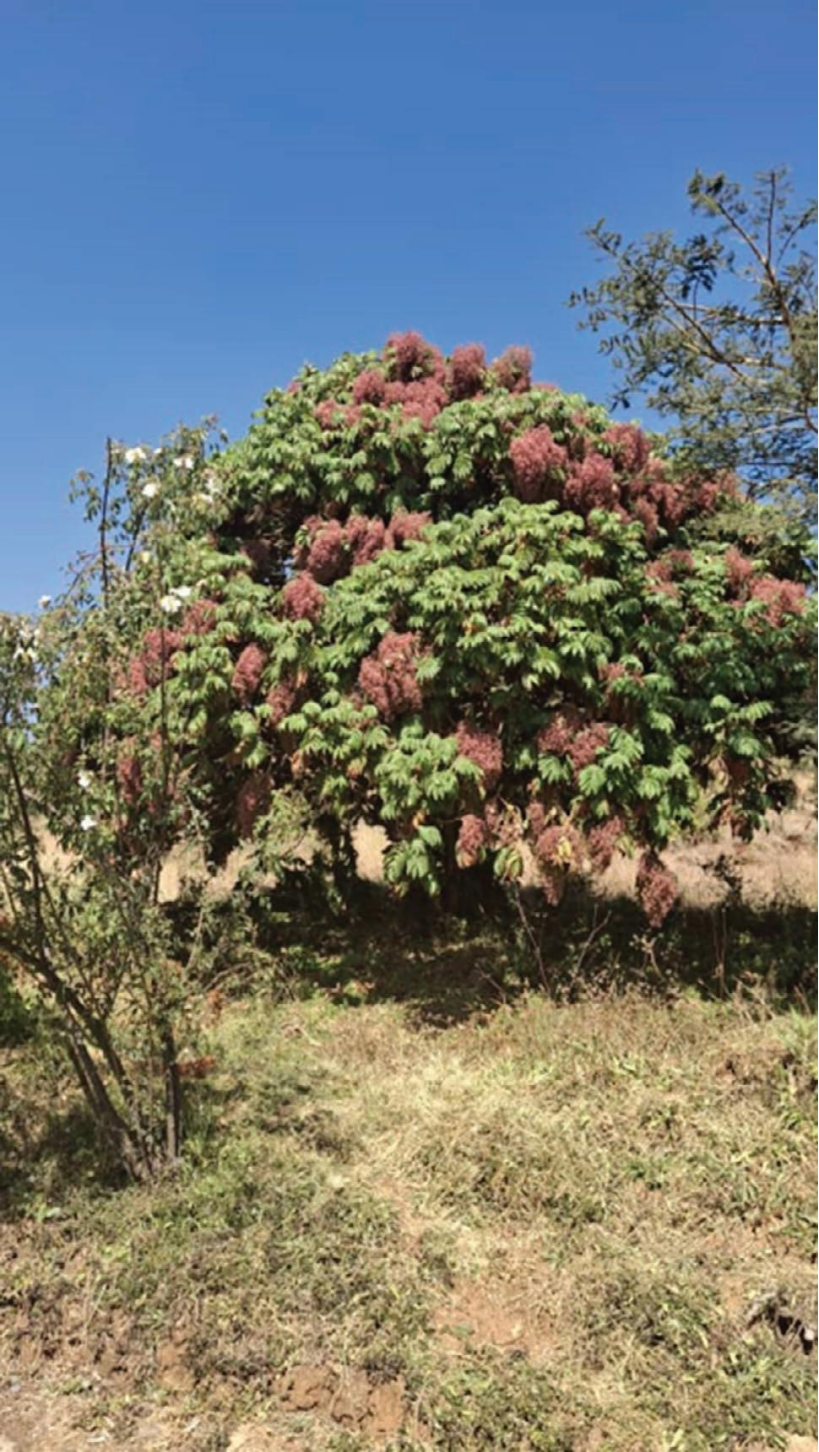
*H. abyssinica* plant at Gulele Botanical Garden, Addis Ababa, Ethiopia (photograph taken by Hirut Basha, October 2024).

Female flowers of *H. abyssinica* were collected from Gulele Botanical Garden in Addis Ababa, Ethiopia (9.0788°N, 38.7211°E) (Figure 1). The specimens were authenticated by Mr. Melaku Wondafrash, a botanist at Addis Ababa University, and a reference specimen (HB002) was deposited at the National Herbarium at Addis Ababa University.

The collected plant material was cleaned to remove contaminants, air‐dried, and mechanically ground. The powdered material was sieved through a 30–40‐mm mesh [15], which was determined to be optimal for extraction. Maceration was performed using 70% ethanol (Loba Chemie, India) with continuous agitation on an orbital shaker (Benchmark Scientific, Inc., United States) for 24 h. After each extraction cycle, the solution was filtered through Whatman No. 1 filter paper (Whatman International Ltd., England), and the remaining plant material was subjected to two additional maceration cycles.

The combined filtrates were concentrated under reduced pressure using a rotary evaporator (BUCHI Labortechnik AG, Switzerland) at 40°C to obtain a crude extract. The dried extract was stored in screw‐capped glass containers under refrigeration until further use [16]. For fractionation, the crude 70% ethanol extract was first defatted with petroleum ether (30 mL × 3) in a separatory funnel until the solvent was colorless. The petroleum ether fraction was separated, dried, and stored. The defatted extract was then partitioned with chloroform (50 mL × 3), and the resulting chloroform fraction was concentrated under reduced pressure and preserved for subsequent analyses.

The percentage yield of the extract was determined using this formula: Yield (*%*) = [Weight of extract free of solvent (g)/weight of dried powder (g)] × 100 Based on this, the % yield of the ethanol extract of *H. abyssinica* flowers, obtained by maceration, was 11.4%. [17].

### 2.2. Snail Collection

Adult *Biomphalaria* species were collected from Tikur Wuha, Hawassa City, Sidama Regional State, Ethiopia, situated within the geographic coordinates of 6°04 ^′^00 ^″^ to 7°10 ^′^00 ^″^ N latitude and 38°26 ^′^30 ^″^ to 38°43 ^′^00 ^″^ E longitude. Snails were harvested from the water using dip‐net scoops and subsequently cleaned to remove any adhering debris. Taxonomic identification was carried out using standard identification keys under the supervision of Professor Seid Tiku of Jimma University. The collected specimens were identified as *Biomphalaria sudanica* based on shell morphology, following an established practical identification guide [18].

### 2.3. Molluscicide Bioassay in the Field

An initial stock solution of 100 mg/L was prepared, from which a series of working solutions was obtained through successive dilutions at the following concentrations: 1, 5, 10, 20, 40, 50, 60, 80, 90, and 100 mg/L. These concentrations were used to evaluate molluscicidal activity against *Biomphalaria* species.

**Figure 2:**
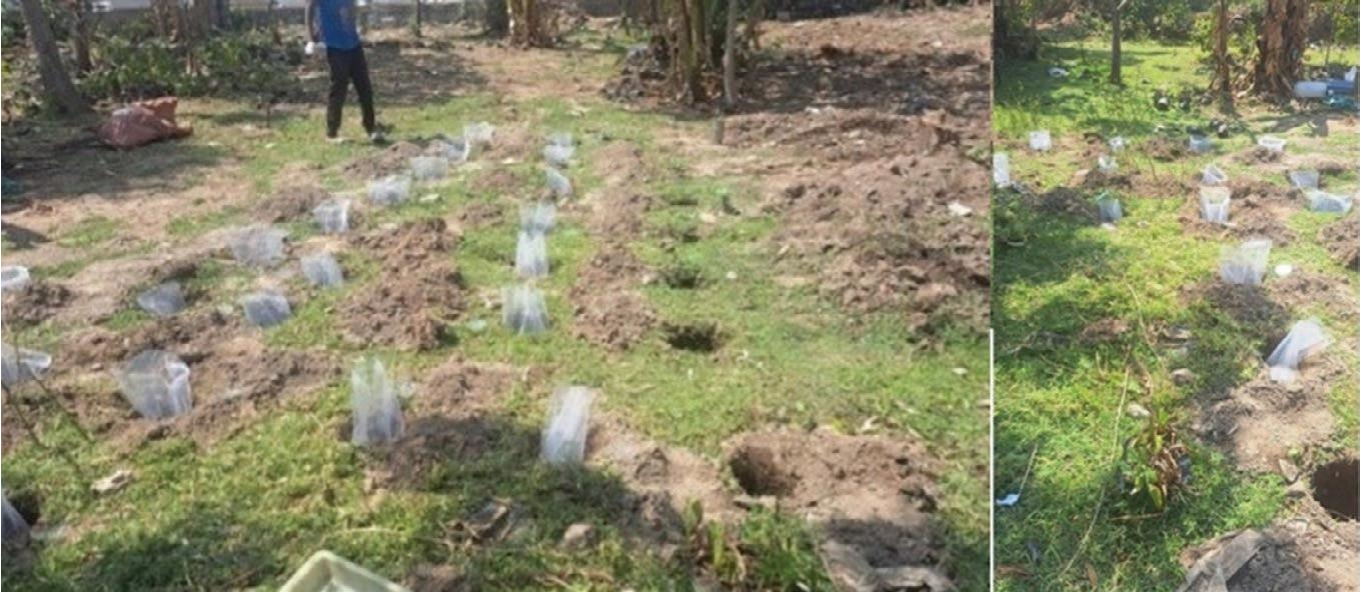
A small‐scale field experiment conducted in plastic‐lined excavated pits near Tikure Wuha in Hawassa City, Sidama Regional State, Ethiopia.

**Figure 3:**
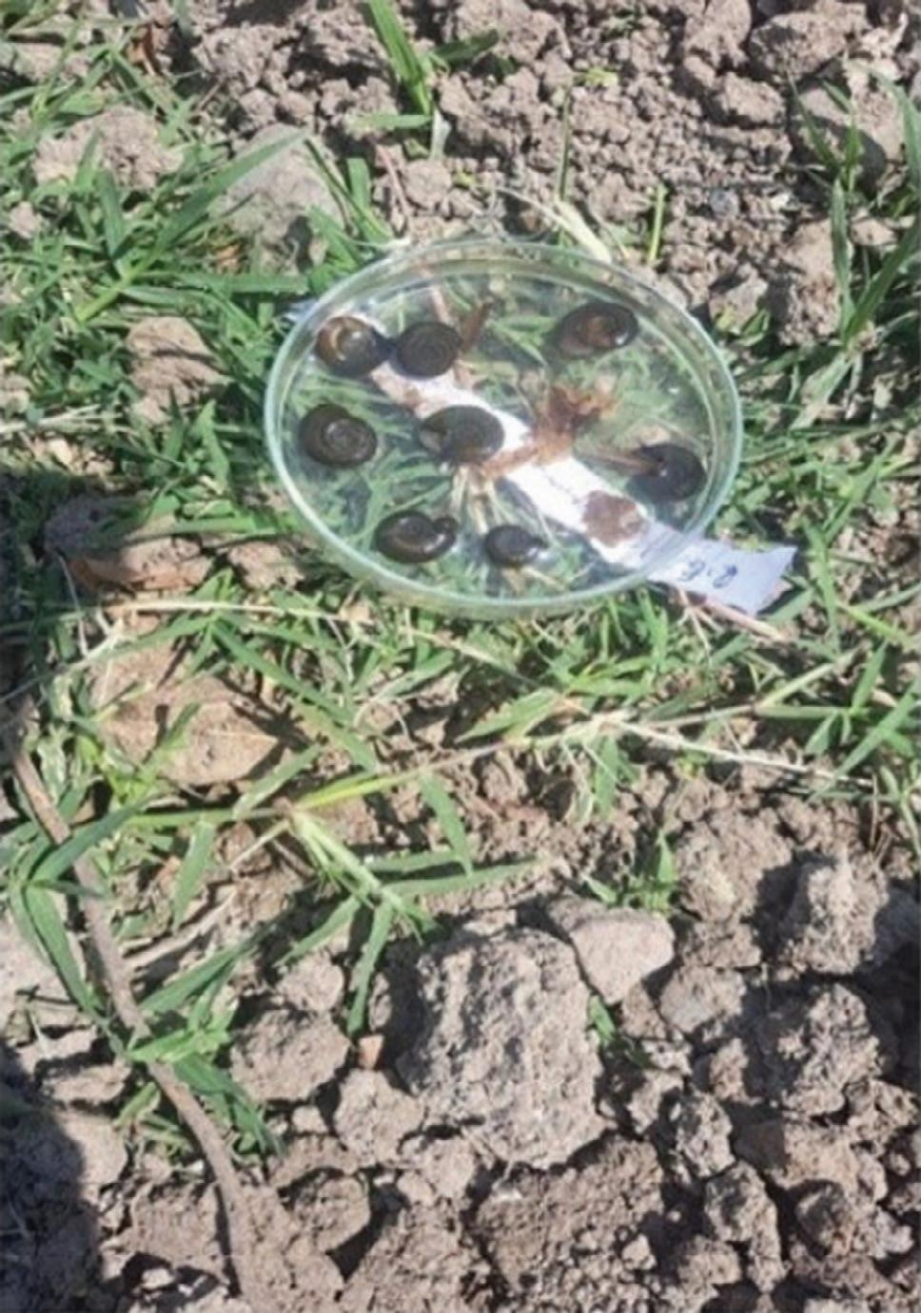
Snails survived after a 24‐h exposure to 20 mg/L of *H. abyssinica* and a 70% ethanol extract.

A small‐scale field experiment was conducted in plastic‐lined excavated pits near Tikure Wuha Lake, Hawassa City, Sidama Regional State, Ethiopia (Figure 2). Each pit measured 0.5 m in length, width, and depth. The experimental design incorporated both negative and positive control groups. River water was utilized as the medium for both the test solutions and controls. Ten snails of uniform size were introduced into each test solution within a pit and exposed for 24, 48, and 72 h (Figure 3). Experiments were performed in triplicate according to WHO guidelines [19]. The river water used had a mean dissolved oxygen (DO) level of 4.89 mg/L, a pH of 7.2, a temperature of 23.1°C, and an electrical conductivity of 1040 *μ*S/cm.

Niclosamide 70% wettable powder was obtained from APEx BIO Technology LLC (United States) and, in line with WHO (2019) recommendations, was used at 1 mg/L as the positive control [19].

### 2.4. Fish Acute Toxicity Test

The acute toxicity of *H. abyssinica* extracts to fish was evaluated in accordance with the Organisation for Economic Co‐operation and Development (OECD) Guideline 203 [20]. Based on the recommendations of this guideline, a minimum of five test concentrations were selected and arranged in a geometric series, with a spacing factor preferably not exceeding 2.2 and, where feasible, maintained within the range of 1.6–1.8, so as to ensure an adequately distributed and reproducible concentration–response relationship.

An initial concentration of 10 mg/L was chosen, as it corresponded to the lowest 72‐h LC_50_ value previously determined for the 70% ethanol extract of *H. abyssinica* flowers in a laboratory molluscicidal activity experiment. A geometric factor of 1.6 was applied to generate the remaining concentrations. The resulting test series comprised 10, 16, 26, 42, 67, and 107 mg/L (ppm). An initial stock solution of 110 mg/L was prepared in a total volume of 45 l. From this, a series of working concentrations of 10, 16, 26, 42, 67, and 107 mg/L was created for fish toxicity testing.

Fish fingerlings with a total length ranging from 3 to 4 cm and a body weight ranging from 5 to 6 g were obtained from the National Fish and Other Aquatic Lives Research Center in Sebeta. The experiment was conducted under static water conditions. Since the experiment was conducted at the same facility where the fish were reared, using water from their natural holding environment, a separate acclimatization period was not required, as the fish were already adapted to the prevailing water conditions.

Seven artificial aquarium tanks were used for the experiment, comprising six tanks assigned to the respective test concentrations and one negative control tank. The control tank contained the test medium only, with no extract added, and served as a baseline reference to assess any mortality occurring independently of the treatment and to ensure that observed effects were solely attributable to the tested extract. Fish were exposed to the six concentrations of the plant extract, with each treatment conducted in duplicate. The study was carried out under controlled greenhouse conditions at the National Fish and Other Aquatic Lives Research Center in Sebeta.

During the 96‐h observation period, fish were monitored every 8 h for signs of erratic swimming, hyperactivity, hyperventilation, decreased responsiveness to visual stimuli, and mortality. Fish were considered dead if they exhibited no response to mechanical stimuli [21].

### 2.5. Test Water Physicochemical Characteristics

**Figure 4:**
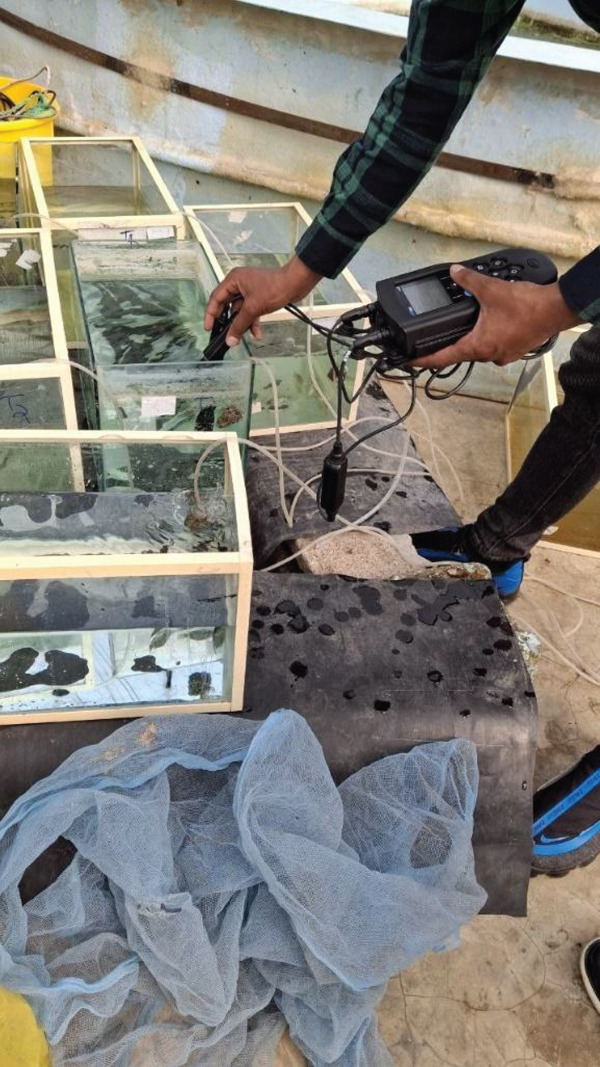
Evaluation of water physicochemical characteristics during the fish toxicity experiment.

DO, temperature, pH, and conductivity of the test water were measured every 24 h using a calibrated multiparameter probe (Figure 4). The probe was calibrated according to the manufacturer′s instructions before each measurement.

### 2.6. Preparation of a Stock Solution for Fish Toxicity

A stock solution with an initial concentration of 110 mg/L was prepared, and a range of working solutions at concentrations of 107, 67, 42, 26, 16, and 10 mg/L were derived from it through serial dilution. These solutions were subsequently used to assess the acute toxicity of *H. abyssinica* on *O. niloticus* fish fingerlings.

### 2.7. Statistical Analysis

Lethal concentrations (LC_50_ and LC_90_) were estimated from mortality data using probit regression analysis in IBM SPSS software. The no observed adverse effect concentration (NOAEC) was defined as the highest concentration at which survival did not differ significantly from the control group. NOAEC values were determined via Dunnett′s t‐test at a 95% confidence level [22].

Mortality data were expressed as response proportions (RP).

### 2.8. Formulas for Fish Toxicity Analysis

First: Mortality data were expressed as follows: RP = number of survivors/total number of exposed.

where RP is the response proportion.

Second: Arcsine transformation

1. For RP values where 0 < RP < 1:

Angle in radians=arcsin√RP

2. Modification of the arcsine when RP = 0:

Angle in radians=arcsin√14/n.

where *n* is the number of animals per treatment replicate.

3. Modification of the arcsine when RP = 1.0:

Angle in radians=1.5708−arcsin√14/n.

where 1.5708 radians = *π*/2, representing the upper bound of the arc sine transformation.

Dunnett′s test was subsequently applied to the arcsine‐transformed mortality data to identify concentrations with no statistically significant difference from the control group at a 95% confidence level.

## 3. Results

The study demonstrated that both 70% ethanol extract and the chloroform fraction of *H. abyssinica* flowers exhibited molluscicidal efficacy against *Biomphalaria* species (Table 1). After 24 h of exposure, the LC_50_ values were 78.77 mg/L for the ethanol extract and 36.49 mg/L for the chloroform fraction (Table 1), whereas the corresponding 24‐h LC_90_ values were 91.74 and 55.93 mg/L, respectively. Niclosamide was used as a positive control at 1 mg/L, achieving complete snail mortality.

**Table 1:** The molluscicidal activities of the flower of *H. abyssinica*, 70% ethanol extract, and chloroform fraction were tested in a small‐scale field trial on *Biomphalaria* snail species.

Exposure time	Extraction			
	70% ethanol		Chloroform fraction	
	LC_50_ with CI	LC_90_ with CI	LC_50_ with CI	LC_90_ with CI
24 h	78.77 (77.73–79.57)	91.74 (86.91–100.5)	36.49(35.31–37.42)	55.93(48.72–74.98)
48 h	78.77 (77.73–79.57)	91.74 (86.91–100.5)	36.21(35.01–37.18)	49.y(44.99–59.83)
72 h	78.34 (77.27–79.12)	88.75 (83.93–95.30)	36.21(35.01–37.18)	49.49(44.99–59.83)

**Figure 5:**
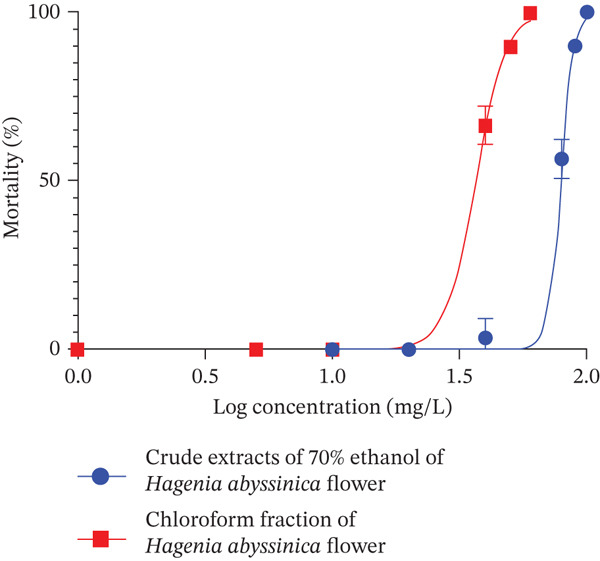
The 24‐h base logarithm of the concentration (mg/L) versus *Biomphalaria* species mortality of 70% ethanol and chloroform fraction of *H. abyssinica* flowers.

Small‐scale field trials demonstrated that both the 70% ethanol and chloroform extracts of *H. abyssinica* flowers exhibited a clear dose–response relationship, with snail mortality increasing proportionally with increasing extract concentration, as illustrated in Figure 5.

### 3.1. Physicochemical Properties

DO levels ranged from 6.15 to 6.89 mg/L, corresponding to more than 65% air saturation. Water temperature varied between 21.1°C and 23.8°C, pH levels ranged from 6.13 to 7.11, and electrical conductivity was measured between 302 and 326 *μ*S/cm (Table 2).

**Table 2:** Measurements of physicochemical properties of the test water in the fish acute toxicity test.

Measurement hours	Test groups	Dissolved (mg/L) (min–max)^#^	Temp. (°C) (min–max)^#^	pH (min–max)^#^	Conductivity (*μ*S/cm) (min–max)^#^
0	Control	6.39	21.2	6.74	315
	Test_1_ to Test_6_∗	6.31–6.39	20.3–21.4	6.61–6.68	308–316

24	Control	6.31	20.5	6.99	312
	Test_1_ to Test_6_∗	6.12–6.44	20.0–22.5	6.13–6.34	302–308

48	Control	6.27	23.8	6.30	324
	Test_1_ to Test_6_∗	6.25–6.56	23.0–24.8	6.29–7.0	305–325

72	Control	6.27	23.8	6.90	324
	Test_1_ to Test_6_∗	6.15–6.67	23.1–24.8	6.29–7.11	312–324

96	Control	6.84	21.7	6.71	316
	Test_1_ to Test_6_∗	6.15–6.89	21.2–21.8	6.40–6.73	306–322

*Note:* Test1 to Test 6∗ refers to the six test concentrations: T1 10mg/L; T2 16 mg/L; T3 26mg/L; T4 42mg/L; T5 67mg/L; and T6 107mg/L (min–max). Number sign “#” refers to the minimum and maximum value of dissolved oxygen, temperature, pH, and conductivity registered between the six test concentrations (i.e., Test1 to Test6∗).

### 3.2. Fish Mortality

**Figure 6:**
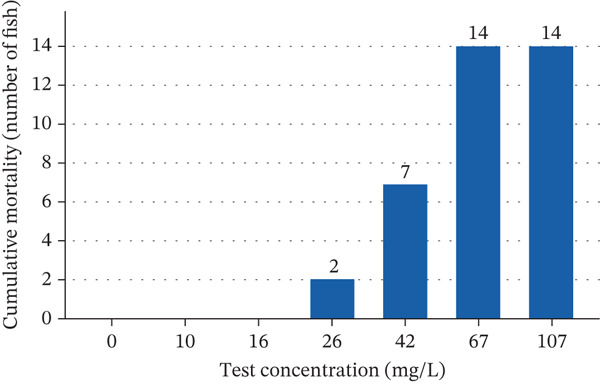
Cumulative mortalities of *O. niloticus* fish fingerlings at different extract concentrations during 96 h of exposure (*n* = 14 per group).

Fish mortality was confirmed by the absence of any observable movement and a lack of response to gentle stimulation of the caudal peduncle. No mortality occurred in the control, 10 and 16 mg/L dilution treatments, whereas complete mortality was observed at 67 and 107 mg/L (Figure 6).

The results indicated that survival at 16 mg/L was statistically indistinguishable from controls (mean = 14; *p* > 0.05, Dunnett′s test), with overlapping confidence intervals and no dose‐dependent trend below this threshold. The first statistically significant reduction in survival occurred at 26 mg/L (mean = 12; *p* < 0.05, Dunnett′s test), representing a 14.3% decline relative to controls. Accordingly, 16 mg/L was designated the NOAEC, representing the highest tested concentration at which no adverse effect on fingerling survival was detectable under the conditions of this study.

The 96‐h LC_50_ value for the 70% ethanol extract from *H. abyssinica* flowers was 38.71 mg/L, with a 95% confidence interval of 32.94–45.83 mg/L, and the LC_90_ was 57.14 mg/L. (Table 3).

**Table 3:** The lethal concentrations and their confidence intervals for 24‐ and 96‐h exposure to a 70% ethanol extract from *H. abyssinica* flowers on Nile tilapia (*Oreochromis niloticus*).

Lethal concentrations	24 h‐concentration (CI at 95%) (in mg/L)	96 h‐ concentration (CI at 95%) (in mg/L)	*χ*2
LC_50_	41.1 (34.9–48.9)	38.7 (32.9‐45.8)	1.57
LC_90_	61.6 (51.2–90.9)	57.1 (47.8‐83.9)	

## 4. Discussions

This study evaluated the molluscicidal efficacy of *H. abyssinica* flower extracts (70% ethanol and chloroform) against *Biomphalaria* species in small‐scale field trials. Acute toxicity to Nile tilapia (*O. niloticus*) was also assessed.

Both extracts demonstrated significant molluscicidal effects against *Biomphalaria* snails under field conditions. The chloroform fraction showed approximately twice the potency of the ethanol extract. This finding is consistent with our previous laboratory study on the molluscicidal activity of *H. abyssinica* flower extracts [11]. The higher potency of the chloroform fraction may be attributed to the solvent′s capacity to dissolve bioactive compounds such as phenols, flavonoids, tannins, and triterpenes [23, 24]. Several of these constituents, particularly flavonoids and triterpenes, are recognized for their molluscicidal properties [25–27]. This likely explains the greater efficacy of the chloroform fraction relative to the 70% ethanol extract.

The 24‐h LC_50_ values for both the 70% ethanol extract and chloroform fraction in field trials were notably higher than those obtained in laboratory experiments (11.93 and 5.52 mg/L, respectively). Similar findings have been reported in previous studies, where LC_50_ and LC_90_ values from laboratory conditions were consistently lower than those from field trials [28]. For example, the LC_90_ of *Galega officinalis* against *Biomphalaria glabrata* was 15 ppm in laboratory assays, while 40 ppm was required to achieve 100% mortality under field conditions [28]. This discrepancy is primarily attributed to differences in water quality between settings. Laboratory experiments used distilled water, whereas field trials were conducted in turbid water containing organic matter. Organic substances likely reduce extract bioavailability by reacting with active compounds, decreasing the amount available to exert molluscicidal activity. Consequently, higher concentrations were required to achieve effective snail mortality under field conditions.

The extracts of *H. abyssinica* exhibited significant molluscicidal activity against *Biomphalaria* species. Efficacy increased proportionally with extract concentration. The time‐dependent reduction in snail survival may be attributed to the slow and sustained release of bioactive compounds. These compounds progressively impaired snail viability over the exposure period [29].

To the best of our knowledge, no previous studies have specifically assessed the toxicity of *H. abyssinica* flower extract on fish. This study, therefore, provides novel data addressing an important knowledge gap regarding the potential effects of these extracts on aquatic organisms.

The LC_50_ value for fish toxicity of *H. abyssinica* flower extract was higher than those reported for other plant‐derived piscicides. For instance, *Sapindus mukorossi* exhibited an LC_50_ of 10 ppm [30], *Jatropha gossypiifolia* showed a piscicidal LC_50_ of 10.49 mg/L [31], and Ph*ytolacca dodecandra* (Endod) had an LC_50_ of 4.4 mg/L [32]. These differences may reflect variation in phytochemical profiles, extraction methods, and species‐specific sensitivity of the test organisms.

The present study determined that the 24‐h LC_50_ of *H. abyssinica* flower extract for Nile tilapia fingerlings was 41.1 mg/L, with a NOAEC of 16 mg/L. These values are notably lower than the molluscicidal 24‐h LC_50_ of 78.4 mg/L reported for the same 70% ethanol extract, indicating that the concentration required to kill 50% of snails is approximately twice that required to produce equivalent mortality in fish. This differential toxicity pattern, where the target organism (snail) tolerates higher concentrations than the nontarget organism (fish) raises important ecological and applied concerns regarding the selective safety of this extract under field conditions.

The greater sensitivity of fish relative to snails may reflect fundamental differences in physiological uptake mechanisms, gill surface exposure, and metabolic detoxification capacity between aquatic vertebrates and gastropod mollusks. Fish, as gill‐breathing vertebrates, are continuously exposed to waterborne compounds across a large absorptive surface area, potentially increasing bioavailability of lipophilic and amphiphilic constituents such as saponins and terpenoids. Saponins, in particular, are known to disrupt gill epithelial integrity and impair osmoregulation in teleost fish, which may explain the disproportionate acute toxicity observed in Nile tilapia fingerlings compared to snails [33]. Additionally, the crude extract contains a complex mixture of bioactive constituents, including alkaloids, phenols, tannins, and flavonoids, whose synergistic interactions may amplify toxicity toward nontarget species beyond what individual compounds would produce in isolation [11].

From an ecological perspective, the selectivity ratio (molluscicidal LC_50_/piscicidal LC_50_ = 78.4/41.1 ≈ 1.91) indicates a narrow safety margin. This means that concentrations needed to effectively control snails are likely to approach or exceed lethal levels for coexisting fish populations. In natural aquatic systems where snails live alongside native fish species, applying this crude extract at concentrations effective against snails could pose a significant risk to nontarget fish, especially juveniles and larvae, which may be more sensitive than the fingerlings tested in the study.

The NOAEC of 16 mg/L established in this study provides a provisional safety threshold. However, this value alone is not enough to ensure ecological safety without additional assessments focused on sublethal effects. Concentrations below the NOAEC may still negatively affect important physiological aspects such as swimming performance, feeding behavior, reproductive success, and immune function in fish, which are not evaluated by acute survival tests. Therefore, future studies should include sublethal biomarkers, such as cortisol response, hematological indices, and histopathological examinations of gill and liver tissues to better evaluate the risk profile of this extract for nontarget fish species.

To enhance the effectiveness of molluscicides while ensuring better safety for nontarget species, future research should focus on bioassay‐guided fractionation. This method allows for the isolation and characterization of the specific components that contribute to molluscicidal activity. Purified or enriched fractions, which have targeted modes of action against mollusks, are generally expected to show lower nonspecific toxicity to vertebrates compared with complex crude extracts [34]. This approach could significantly improve the selectivity ratio and aid in the development of a more environmentally friendly botanical molluscicide derived from *H. abyssinica.*

DO concentrations ranged from 6.15 to 6.89 mg/L across all treatments. These values met the U.S. Environmental Protection Agency′s recommendation that DO levels should not fall below 6.0 mg/L for cold‐water species [21]. Water temperatures ranged from 21.1°C to 23.8°C, remaining within recommended limits. The U.S. Environmental Protection Agency specifies that test temperatures should not fluctuate by more than 3°C between maximum and minimum values during the test period [22]. All physicochemical parameters were therefore maintained within acceptable ranges throughout the study.

One limitation of this study is that, although the chloroform extract demonstrated significant molluscicidal activity, it could not be included in the fish toxicity assay due to insufficient quantities of the extract available at the time. Fish toxicity testing requires higher concentrations, and the limited amount of chloroform extract obtained was inadequate for this purpose. Additionally, since *H. abyssinica* is a seasonally available plant, further collection and re‐extraction were not feasible within the project′s timeframe.

## 5. Conclusion

The 70% ethanol extract and chloroform fraction of *H. abyssinica* flowers exhibited strong molluscicidal activity against *Biomphalaria* snails. Both extracts show potential as effective molluscicides. Acute toxicity to fish was dose‐dependent. Survival was unaffected at concentrations at or below the NOAEC of 16 mg/L but declined significantly at higher concentrations. Nevertheless, this value by itself is insufficient to guarantee ecological safety unless further evaluations concentrate on sublethal effects. Levels below the NOAEC could still adversely impact critical physiological factors, which are not assessed through acute survival tests. This finding highlights the importance of careful dosing in field applications to minimize risk to nontarget species.

## 6. Recommendation

Future studies should evaluate isolated molluscicidal compounds to determine whether purification can maintain snail toxicity while reducing fish toxicity. Further investigations across diverse snail habitats are also warranted to confirm the effectiveness and practical applicability of these extracts under varying environmental conditions. Furthermore, future studies should incorporate chloroform extracts in fish toxicity evaluations to provide a more comprehensive toxicological profile.

## Author Contributions

H.B., H.M., and H.T. conceptualized and designed the study. H.B. drafted the manuscript and performed the data analysis. All authors (H.B., A.D., M.E., M.M., H.T., and H.M.) participated in the experimental work, contributed to data analysis and interpretation, and critically revised the manuscript for important intellectual content.

## Funding

This work is supported by the Armauer Hansen Research Institute, Addis Ababa, Ethiopia.

## Disclosure

The results/data/figures in this manuscript have not been published elsewhere, nor are they under consideration (from you or one of your contributing authors) by another publisher. I confirm the corresponding author has read the journal policies and submit this manuscript in accordance with those policies. All of the material is owned by the authors, and/or no permissions are required. All the generated data in this article are included in the manuscript.

## Ethics Statement

The Ethiopian Public Health Institute Institutional Review Board has granted ethical approval. The protocol number assigned to this approval is EPHI‐IRB‐513‐2023.

## Conflicts of Interest

The authors declare no conflicts of interest.

## Data Availability

The data that support the findings of this study are available from the corresponding author upon reasonable request.
